# Homicides in Peru: the importance of accurate and timely recording of causes of death

**DOI:** 10.17843/rpmesp.2025.423.14947

**Published:** 2025-08-12

**Authors:** Akram Hernández-Vásquez, Luis Ttito-Paricahua, Richard Asmat-Condormango, Judith Quiñones-Inga

**Affiliations:** 1 Universidad San Ignacio de Loyola, Vicerrectorado de Investigación, Centro de Excelencia en Investigaciones Económicas y Sociales en Salud, Vicerrectorado de Investigación, Universidad San Ignacio de Loyola, Lima, Peru. Universidad San Ignacio de Loyola Universidad San Ignacio de Loyola Vicerrectorado de Investigación, Centro de Excelencia en Investigaciones Económicas y Sociales en Salud Vicerrectorado de Investigación, Universidad San Ignacio de Loyola Lima Peru; 2 Programa de Maestría en Salud Pública, Universidad Científica del Sur, Lima, Peru. Universidad Científica del Sur Programa de Maestría en Salud Pública Universidad Científica del Sur Lima Peru; 3 Instituto Nacional de Seguridad Social para Jubilados y Pensionados de Argentina, Buenos Aires, Argentina. Instituto Nacional de Seguridad Social para Jubilados y Pensionados de Argentina Buenos Aires Argentina; 4 Programa de Maestría en Epidemiología Clínica y Bioestadística, Universidad Científica del Sur, Lima, Peru. Universidad Científica del Sur Programa de Maestría en Epidemiología Clínica y Bioestadística Universidad Científica del Sur Lima Peru

Mr. Editor. In recent years, Peru has experienced an increase in the number of homicides ^1^, which has triggered a deep crisis in citizen security and public health. The precise identification of the specific causes of these homicides is essential for developing effective interventions. In this regard, medico-legal autopsies are a fundamental tool, as they allow for the establishment of the cause, manner, and circumstances of death ^2^, aspects that are essential for forensic investigations and judicial proceedings.

Currently, the National Death Information System (SINADEF) is an online electronic registry that allows for the medical certification of a death within hours of its occurrence ^3^. Although it is not the only source of information on deaths in the country, its implementation has been essential for obtaining timely data that contributes to decision-making in public policy. Using this system, deaths from external causes are certified by the medical examiner or the physician designated by law, who must correctly complete the death certificate. However, if the records on homicides have omissions, inaccuracies, or errors in the coding according to the International Classification of Diseases (ICD-10) or in the description of the cause of death, the ability to adequately quantify the problem would be compromised ^4^. This would hinder the creation of accurate situational analyses and negatively affect the response capacity of authorities to the growing phenomenon of violence in the country.

Given this scenario, a study was conducted with the objectives of describing the evolution of homicides in Peru between 2017 and 2024, evaluating the quality of ICD-10 coding, and characterizing the injury mechanisms of death. The external causes listed on the death certificates of all cases with an autopsy were reclassified through a detailed review of each of the causes of death. The original descriptions contained in causes A through F were hierarchically categorized into four groups: deaths by firearm, bladed weapon, asphyxia, and other causes. This reclassification was carried out using specific forensic terms identified by two authors (AHV and LTP) and validated by a third author specializing in legal medicine (RAC). As an example, when terms, mechanisms, or writing errors related to a firearm (“bala” [bullet], “PAF” [firearm projectile], “arna de fuego” [firearm mispelled], “proyectikl” [projectile mispelled], “perforo contusa” [perforating blunt], among others) were found, the death was classified under this category. The same was done successively with the other categories. The terms used for classification are in the Supplementary Material. Additionally, a freely accessible interactive dashboard was developed that shows the evolution of homicides and injury mechanisms. Python and its Streamlit library for interactive data visualization were used for processing and statistical analysis.

Between 2017 and 2024, 10,086 deaths classified as homicides that underwent an autopsy were recorded. The results show a sustained increase in the absolute number of homicides during this period. Of the total deaths, 8713 (86.4%) were male. Figure 1A shows a high percentage of cases where “no record” is entered in the ICD-10 code for the causes of death. Regarding the recorded ICD-10 codes, 2169 were registered in Cause A (21.5%), 1734 in Cause B, 1237 in Cause C, 252 in Cause D, 32 in Cause E, and only 1 code in Cause F. Figure 1B shows a progressive increase in the number of homicides recorded in the country, as well as the classification of deaths according to the identified terms, with deaths by firearm predominating (66.7%), which increased from 357 cases (54.7%) in 2017 to 1532 cases (75.4%) in 2024. Finally, the word cloud (Figure 1C) corresponding to the causes of death classified as “Other causes” shows a predominance of the term “no record,” accompanied by others such as “traumatism,” “fracture,” “hemorrhage,” “penetrating,” “laceration,” and “cranium.” These terms suggest a diversity of injury mechanisms and, in some cases, the difficulty in precisely determining the specific cause of death.

**Figure 1:**
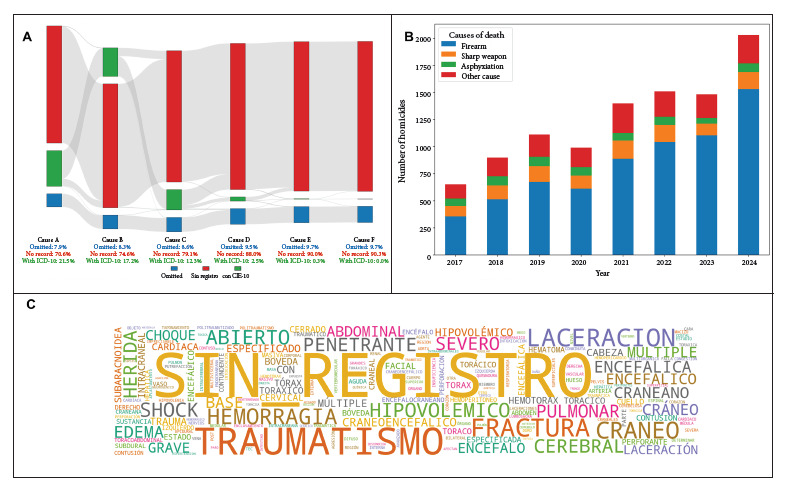
A. Distribution of ICD-10 code records for each cause of death, classified as “Omitted” (when the field was empty), “No record” (when the text “No record” was found), or “With ICD-10” (when an ICD-10 code was found). B. Evolution of the number of homicides in Peru between 2017 and 2024, classified by firearm, bladed weapon, asphyxia, or other causes. C. Word cloud for the “Other cause” of death category, which was obtained by combining the original descriptions of causes A through F into a single field and excluding the terms “EN”, “EL”, “LA”, “LAS”, “DE”, “POR”, “QUE”, “Y”, “DEL”, “E”, “X”, “UNA”, “MÁS”, “SIN”, “SUB”, “MAS”, “O”, “LOS”.

In this context, it is essential to continue strengthening SINADEF’s capacity to improve the quality of records, including periodic and systematic training of professionals responsible for certifying deaths from external causes. This would reduce classification errors and improve the accuracy of ICD-10 coding, facilitating more precise and useful situational analyses for decision-making. Likewise, compliance with the protocols and regulatory documents established by the Institute of Legal Medicine and Forensic Sciences ^5^ and the Ministry of Health ^(^^6^^,^^7^ must be ensured to correctly guide the completion of the death certificate by recording the ICD-10 codes for the causes of death and avoid ambiguities or erroneous interpretations. Only through adequate and reliable information can effective public policies be designed to successfully confront the growing violence in the country, allowing the State to fulfill its duty to protect life and guarantee citizen security.

Finally, we are making an interactive dashboard available to visualize the annual, monthly, and weekly evolution of homicides in Peru. This dashboard will be kept updated to contribute to the surveillance and sustained analysis of this problem. It is available at the following electronic address: https://homicidios-sinadef.streamlit.app/.

## References

[1] (2025). Número de homicidios en Perú 2025.

[2] Hanzlick RL (2015). The "Value-Added" Forensic Autopsy Public Health, Other Uses, and Relevance to Forensic Pathology's Future. Acad Forensic Pathol.

[3] Vargas-Herrera J, Miranda-Monzón J, Lopez-Wong L, Miki-Ohno J (2022). La cobertura de muertes con certificación médica en el Perú, 2012-2019. An Fac Med.

[4] Palomo-Rando JL, Ramos-Medina V, de la Cruz Mera E, López-Calvo AM (2010). Diagnóstico del origen y la causa de la muerte después de la autopsia médico-legal (Parte I). Cuad Med Forense.

[5] Ministerio Público (2007). Resolución de la Fiscalía de la Nación N° 129-2007-MP-FN. Manual Procedimientos Tanatológicos forenses y servicios complementarios y Manual de Procedimientos de la Diligencia de Levantamiento de Cadáver.

[6] Ministerio de Salud (2016). Resolución Ministerial N° 280-2016/MINSA. Aprobar la Directiva Administrativa N°216-MINSA/OGTI-V.01: Directiva Administrativa que establece el Procedimiento para la Certificación de las Defunciones.

[7] Ministerio de Salud (2018). Guía técnica para el correcto llenado del Certificado de Defunción.

