# Supplementary material for: Homicides in Peru: the importance of accurate and timely recording of causes of death
Source: Rev Peru Med Exp Salud Publica. 2025 Aug 12;42(3):328–30. doi: 10.17843/rpmesp.2025.423.14947 (PMC12679969; doi:10.17843/rpmesp.2025.423.14947)
Supplement: Supplementary material. — Available in the electronic version of the RPMESP. [file rpmesp-42-03-14947-s001.docx]

**Material suplementario**

**Anexo 1.** La clasificación de las causas de muerte incluyó una serie de términos extraídos de la descripción de las causas de muerte de A - F que incluye variantes ortográficas, sinónimos y errores. Cada registro de homicidios se asignó a una de las siguientes categorías:

1. Arma de fuego
   1. Se consideraron los siguientes términos: “arma de fuego”, “arma de fuefo”, “arma de fueego”, “arna de fuego”, “proyectíl”, “proyectil”, “proyectiles”, “proyectikl”, “poyectil”, “paf”, “bala”, “porbala”, “armade”, “perdigon”, “perdigones”, “disparo”, “disparado”, “percutado”, “p.a.f.”, “pafen”, “p.a.f”, “p. a. f.”, “perforo contusas”, “perforo contusa”, “perforo‑contusa”, “perforo‑contusas”, “perforo – contusas”, “perforo – contusa”, “perforocontusas”, “perforocontusa”, “perfocontusas”, “perforo‑contuso”, “perforo contuso”, “peroforo‑contusas”, “perforo – contuso”, “perforo –contusa”, “ferforo – contusas”, “perforocontuss”, “contuso perforante”, “orificio de entrada”, “heridas multiples por fdf”, “herida perforante”, “heridas perforantes”, “lesion perforante”, “curso perforante”, “curso perforantes”, “curso penetrante”.
   2. Además, cualquier registro de homicidio que contuviera simultáneamente los términos “perforante” y “penetrante” (incluidos plurales y variantes).
2. Arma blanca
   1. Se consideraron los siguientes términos relacionados con objetos cortantes o punzantes, tales como: “arma blanca”, “arma blanca”, “punzocortante”, “punzocortarte”, “punzo cortante”, “punzo‑cortante”, “punzocortantes”, “punzo – cortante”, “punzo – cortantes”, “punzo‑cortantes”, “punza – cortante”, “lesion cortante”, “lesión cortante”, “punzopenetrante”, “punzo‑penetrante”, “punzo – penetrante”, “punzo penetrante”, “punza cortante”, “punzocortopenetrante”, “punzocortoperforante”, “punzo‑corto‑penetrante”, “punzo corto penetrante”, “ponzo cortantes”, “punzo cortante penetrante”, “punzo cortante penetrantes”, “punzocortante penetrante”, “puncortantes”, “punzocorto penetrante”, “punzo‑corto penetrante”, “corto‑punzo‑penetrante”, “punzo‑ penetrante”, “corto punzante”, “cortantes y penetrantes”, “punzo‑corzo‑penetrante”, “corto punzantes”, “contusocortante”, “contusocortantes”, “contuso‑cortante”, “contusas cortantes”, “corto penetrante”, “corto penetrantes”, “cortopenetrante”, “cortopenetrantes”, “corto‑penetrante”, “contuso‑cortantes”, “contuso cortante”, “objeto cortante”, “degüello”, “deguello”, “degúello”, “degullo”, “degollamiento”, “herida cortante”, “decapitación”, “punzante”, “punta – filo – hoja”, “decapitacion”, “punta y‑o filo”, “punta y filo”, “punta yo filo”, “botella rota”, “seccion de traquea y vasos de cuello”, “seccion de laringe y grandes vasos”, “sección total de laringe y de vasos sanguineos”, “seccionamiento de paquete vasculo nervioso cevical”, “herdas cortantes”, “heridas cortantes”, “cuchillo”, “filo y peso”, “objeto con uso cortante”, “traumatismos cortantes”, “herida transfixiante en cuello”.
3. Asfixia
4. Se contemplaron los siguientes términos: “estrangulación”, “estrangulamiento”, “estrangulacion”, “asfixia”, “asfiixia”, “extrangulamiento”, “asfixias”, “ahorcamiento”, “ahorcadura”, “sofocacion”, “ahogamiento”.
5. Otra causa
   1. Los homicidios cuya descripción de causas no coincidió con ninguno de los términos anteriores se clasificaron como “Otra causa”.
