# Supplementary material for: Homicides in Peru: the importance of accurate and timely recording of causes of death
Source: Rev Peru Med Exp Salud Publica. 2025 Aug 12;42(3):328–30. doi: 10.17843/rpmesp.2025.423.14947 (PMC12679969; doi:10.17843/rpmesp.2025.423.14947)
Supplement: Translation

# Translation: Homicidios en el Perú: importancia de un registro preciso y oportuno de las causas de muerte

**Authors:** Akram Hernández-Vásquez, Luis Ttito-Paricahua, Richard Asmat-Condormango, Judith Quiñones-Inga

**Affiliations:** 1 Center of Excellence in Economic and Social Research in Health, Vice-Rectorate for Research, Universidad San Ignacio de Loyola, Lima, Perú.; 2 Master’s Program in Public Health, Universidad Científica del Sur, Lima, Perú.; 3 National Institute of Social Security for Retirees and Pensioners of Argentina, Buenos Aires, Argentina.; 4 Master’s Program in Clinical Epidemiology and Biostatistics, Universidad Científica del Sur, Lima, Perú.

Sr. Editor. En los últimos años, el Perú ha registrado un incremento en el número de homicidios ^1^, lo que ha desencadenado una profunda crisis en materia de seguridad ciudadana y salud pública. La identificación precisa de las causas específicas de estos homicidios es indispensable para desarrollar intervenciones efectivas. En tal sentido, las necropsias de ley constituyen una herramienta fundamental, al permitir establecer la causa, la forma y circunstancias de muerte ^2^, aspectos esenciales para las investigaciones forenses y los procedimientos judiciales.

Actualmente, el Sistema Informático Nacional de Defunciones (SINADEF) es un registro electrónico en línea que permite la certificación médica de una muerte a pocas horas de su ocurrencia ^3^. Si bien no es la única fuente de información sobre defunciones en el país, su implementación ha sido esencial para la obtención oportuna de datos que contribuyen a la toma de decisiones en políticas pública. En este sistema, las muertes por causa externa son certificadas por el médico legista o por el médico designado por ley, quienes deben realizar un correcto llenado del certificado de defunción. No obstante, si los registros sobre homicidios presentaran omisiones, imprecisiones o errores en la codificación según la Clasificación Internacional de Enfermedades (CIE-10) o de la descripción de la causa de muerte, se vería comprometida la capacidad de cuantificar adecuadamente el problema ^4^. Ello dificultaría la realización de análisis situacionales precisos y afectaría negativamente la capacidad de respuesta de las autoridades frente al creciente fenómeno de violencia en el país.

Ante este escenario, se realizó un estudio cuyos objetivos fueron describir la evolución de los homicidios en el Perú entre 2017 y 2024, evaluar la calidad de la codificación CIE-10, y caracterizar los mecanismos lesionales de muerte. Para ello, se reclasificaron las causas externas consignadas en los certificados de defunción de todos los casos con necropsia, mediante una revisión detallada de cada una de las causas de muerte. Las descripciones originales contenidas en las causas A hasta la F fueron categorizadas jerárquicamente en cuatro grupos: muertes por arma de fuego, arma blanca, asfixia y otras causas. Esta reclasificación se realizó empleando términos forenses específicos que fueron identificados por dos de los autores (AHV y LTP) y validados por un tercer autor especialista en medicina legal (RAC). Así, por ejemplo, cuando se encontraron términos, mecanismos, o errores de redacción relacionados con un arma de fuego (“bala”, “PAF”, “arna de fuego”, “proyectikl”, “perforo contusa”, entre otros), se clasificó la muerte bajo esta categoría, y lo mismo se hizo sucesivamente con las otras categorías. Los términos usados para la clasificación se encuentran en el Material Suplementario. Asimismo, se desarrolló un tablero interactivo de acceso libre que muestra la evolución de los homicidios y los mecanismos lesionales. Se utilizó Python para el procesamiento, análisis estadístico y su librería Streamlit para la visualización interactiva de los datos.

Entre 2017 y 2024 se registraron 10 086 muertes clasificadas como homicidios que contaron con la realización de una necropsia. Los resultados muestran un aumento sostenido en el número absoluto de homicidios durante este periodo. Del total de muertes, 8713 (86,4%) correspondieron a hombres. La Figura 1A muestra que, existe un alto porcentaje de casos donde se consigna “sin registro” en el código CIE-10 de las causas de muerte. En cuanto a los códigos CIE-10 consignados, se registraron 2169 en la Causa A (21,5%), 1734 en la Causa B, 1237 en la Causa C, 252 en la Causa D, 32 en la Causa E, y solo 1 código en la Causa F. En la Figura 1B se evidencia un incremento progresivo en el número de homicidios registrados en el país, así como la clasificación de las muertes según los términos identificados, predominando las muertes por arma de fuego (66,7%), las cuales aumentaron de 357 casos (54,7%) en 2017 a 1532 casos (75,4%) en 2024. Finalmente, la nube de palabras (Figura 1C) correspondiente a las causas de muerte clasificadas como “Otras causas” muestra un predominio del término “sin registro”, acompañado de otros como “traumatismo”, “fractura”, “hemorragia”, “penetrante”, “laceracion” y “craneo”. Estos términos sugieren una diversidad de mecanismos lesionales y en algunos casos, la dificultad para determinar con precisión la causa específica de muerte.

**Figura 1:** A. Distribución de los registros de códigos CIE-10 para cada causa de muerte, clasificados como “Omitida” (cuando se encontraba el campo vacío), “Sin registro” (cuando se encontraba el texto “Sin registro”) o “Con CIE-10” (cuando se encontraba un código CIE-10). B. Evolución del número de homicidios en el Perú entre 2017 y 2024, clasificados según arma de fuego, arma blanca, asfixia u otras causas. C. Nube de palabras correspondiente a la categoría “Otra causa” de muerte, que se obtuvo luego de la unión en un solo campo de las descripciones originales de las causas A hasta F y se excluyeron los términos “EN”, “EL”, “LA”, “LAS”, “DE”, “POR”, “QUE”, “Y”, “DEL”, “E”, “X”, “UNA”, “MÁS”, “SIN”, “SUB”, “MAS”, “O”, “LOS”.

En este contexto, resulta imprescindible seguir fortaleciendo la capacidad del SINADEF para mejorar la calidad de los registros, incluyendo la capacitación periódica y sistemática de los profesionales encargados de certificar las muertes por causa externa. Esto permitiría reducir errores de clasificación y mejorar la precisión en la codificación según el CIE-10, facilitando análisis situacionales más precisos y útiles para la toma de decisiones. Asimismo, se debe asegurar el cumplimiento de los protocolos y documentos normativos establecidos por el Instituto de Medicina Legal y Ciencias Forenses ^5^ y el Ministerio de Salud ^6^^,^^7^, para guíar correctamente el llenado del certificado de defunción mediante el registro de los códigos CIE-10 de las causas de muerte y así evitar ambigüedades o interpretaciones erróneas. Solo mediante información adecuada y confiable se podrán diseñar políticas públicas efectivas para enfrentar exitosamente la creciente violencia en el país, permitiendo al Estado cumplir con su deber de protección a la vida y garantizar la seguridad ciudadana.

Finalmente, ponemos a disposición un tablero interactivo que permite visualizar la evolución anual, mensual y semanal de los homicidios en el Perú, el cual se mantendrá actualizado para contribuir con la vigilancia y el análisis sostenido de esta problemática, que está disponible en la siguiente dirección electrónica: https://homicidios-sinadef.streamlit.app/.
